# Supplementary material for: Shape and structural relaxation of colloidal tactoids
Source: Nat Commun. 2022 May 19;13:2778. doi: 10.1038/s41467-022-30123-y (PMC9120485; doi:10.1038/s41467-022-30123-y)
Supplement: Supplementary file 1 — Supplementary information [file 41467_2022_30123_MOESM1_ESM.pdf]

Supplementary Information for:

Shape and structural relaxation of colloidal tactoids

Hamed Almohammadi[1]†, Sayyed Ahmad Khadem[2,3]†, Massimo Bagnani[1], Alejandro D. Rey[2,3], Raffaele Mezzenga[1,4*]

[1]Department of Health Sciences and Technology, ETH Zurich, Zurich, Switzerland
[2]Department of Chemical Engineering, McGill University, Montreal, QC, Canada
[3]Quebec Centre for Advanced Materials, Canada (QCAM/CQMF), Montreal, QC, Canada
[4]Department of Materials, ETH Zurich, Zurich, Switzerland

†These authors contributed equally to this work.
*Correspondence to: raffaele.mezzenga@hest.ethz.ch

Supplementary Fig. 1

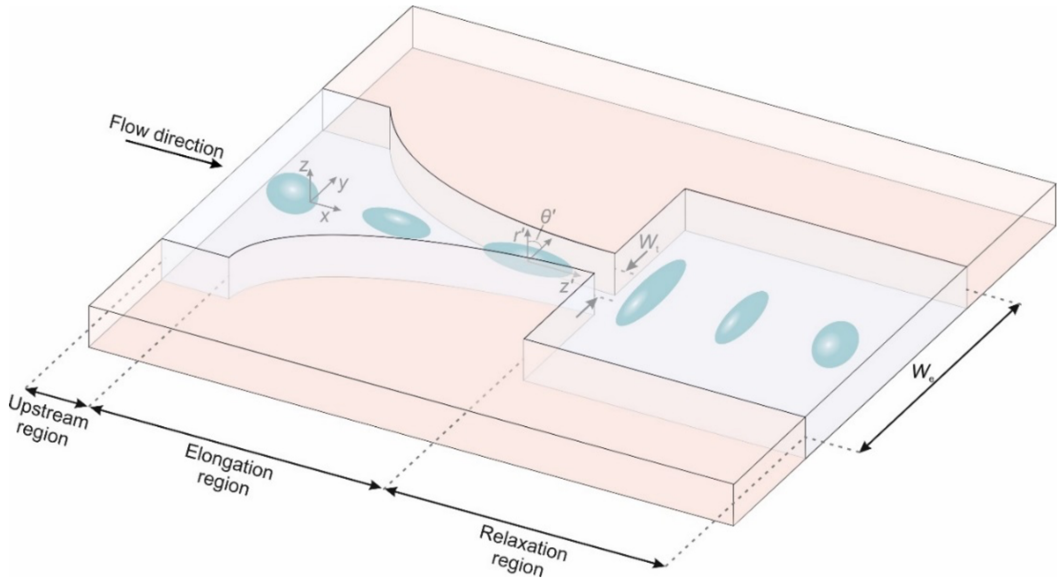

Supplementary Fig. 1 | Microfluidic system used to study the tactoids elongation and relaxation. A liquid crystalline suspension with a concentration within the isotropic–nematic coexistence region is injected to the microfluidic system, allowing to form tactoids with various volumes at upstream region. Tactoids travel in flow direction and are elongated in the elongation region in the flow direction. Right at the beginning of the relaxation region, tactoids get elongated again but in the perpendicular direction with respect to the flow direction and undergo relaxation. The coordinate *x*-*y*-*z* is located on the center line of the channel at the beginning of contraction zone. The coordinate *r′*-*z′*-*θ′* is located at the center of the tactoid. The geometry of the contraction zone is defined as $w(x) = x_1/(x_2+x)$, where $x_1 = l_c w_e w_t/[\ (w_e-w_t)]$, $x_2 = l_c w_t/(w_e-w_t)$, $w(x)$ is the width of the elongation region, l_e is the length of extension region, l_c is the length of elongation region, l_u is the length of upstream, h is the height of the channel, w_e is the upstream or downstream (extension region) width, and w_t is the throat width."

Supplementary Fig. 2

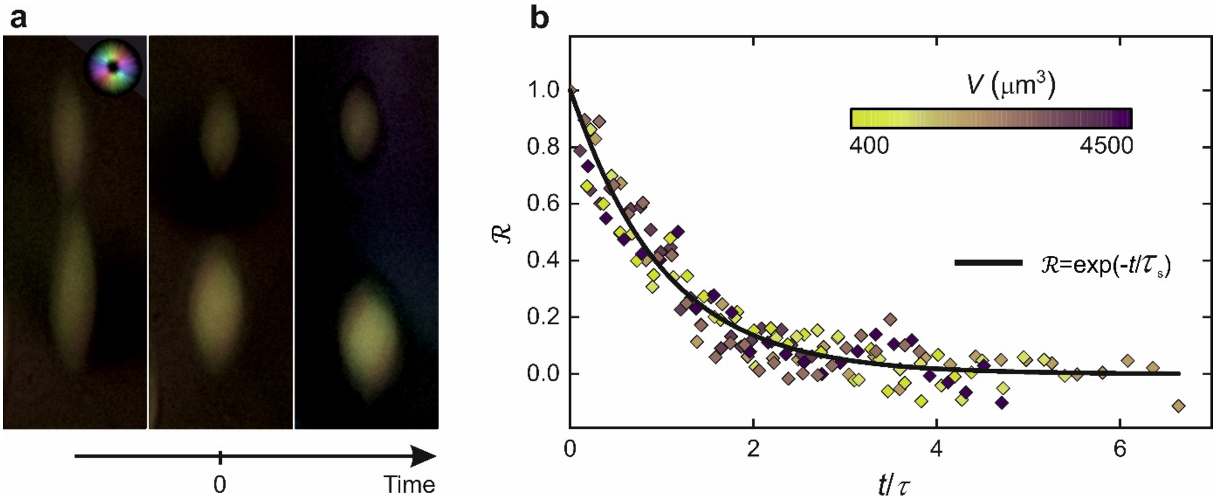

Supplementary Fig. 2 | Shape relaxation of amyloid fibril tactoids following the breakup event. a, The LC (liquid crystal)-PolScope images capturing the breakup of the tactoids that is followed by relaxation of the resultant two tactoids. The time zero here is defined as the moment when the two resultant tactoids are disconnected. **b**, Experimental results, showing that shape relaxation of the tactoids, resulted from the breakup event, follow single exponential decay similar to the initially extended tactoids relaxation.

Supplementary Fig. 3

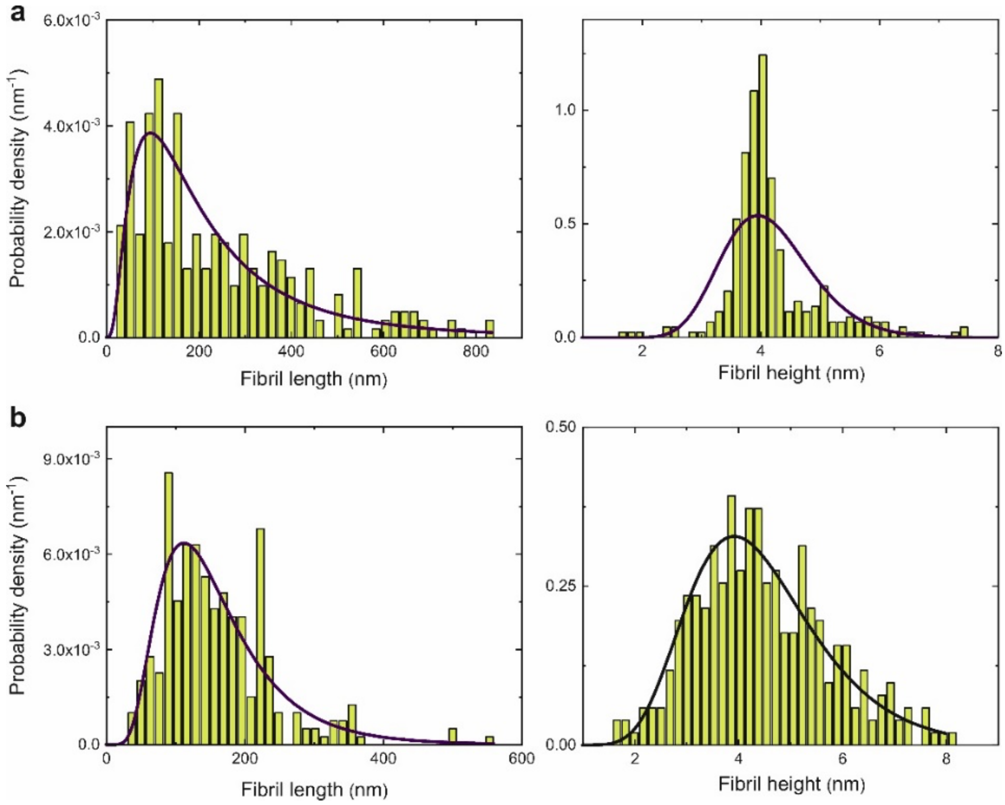

Supplementary Fig. 3 | Length and height distributions of liquid crystalline systems. Lognormal distribution fitted to data (n = 300) as shown with solid lines. **a,** The length and height distributions of BLG II. **b,** The length and height distributions of SCNC.

Supplementary Fig. 4

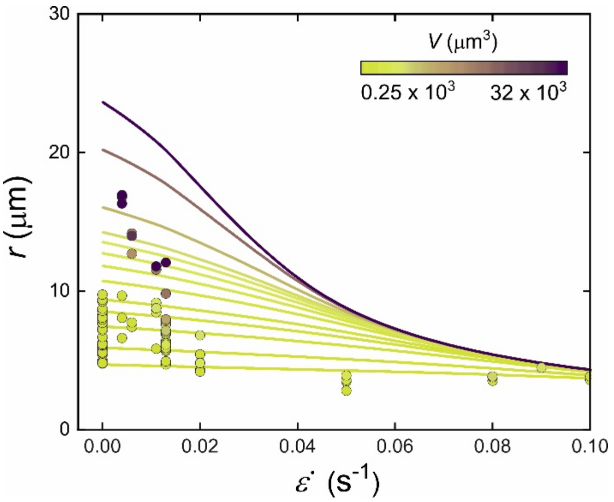

Supplementary Fig. 4 | Maximum deformation of tactoids under various extension rate. The theory (lines) and the experimental data (symbols) predict the short axis of the tactoids r under various extension rate. The data corresponding to the zero shear where r is equal to its equilibrium value are obtained from the tactoids at equilibrium condition in a cuvette. Note that since the modeling (equation 4) assumes the homogenous internal configuration for the tactoids under the shear rate, at zero extension rate only the data of the tactoids that hold homogenous configuration at equilibrium (or at zero shear extension rate) are presented.

Supplementary Fig. 5

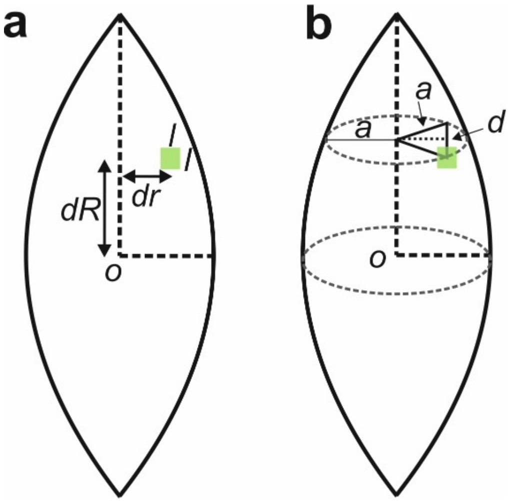

Supplementary Fig. 5 | Schematic showing how the thickness behind every pixel is obtained. a, 2D schematic of the tactoids as it is seen in retardance images. The green square is a schematic of a pixel that is seen in the retardance images. **b**, 3D schematic of the tactoids used to calculate the thickness *d* of the sample behind every pixel.

Supplementary Fig. 6

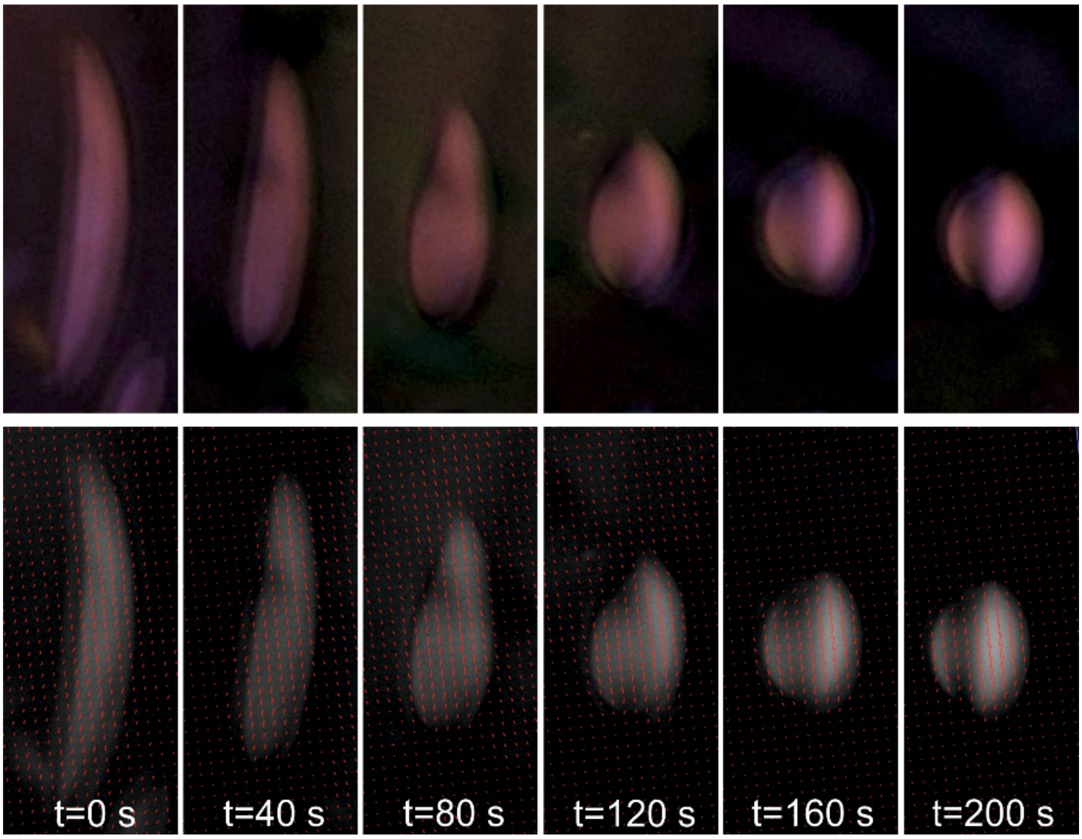

Supplementary Fig. 6 | Shape and director field relaxation of the cholesteric tactoid. The LC (liquid crystal)-PolScope images showing the relaxation of the of amyloid fibril liquid crystalline tactoid. The director field data shows that the change/rotation in the director field extend to the end of the relaxation process and shows significant changes at the late stage.

Supplementary Fig. 7

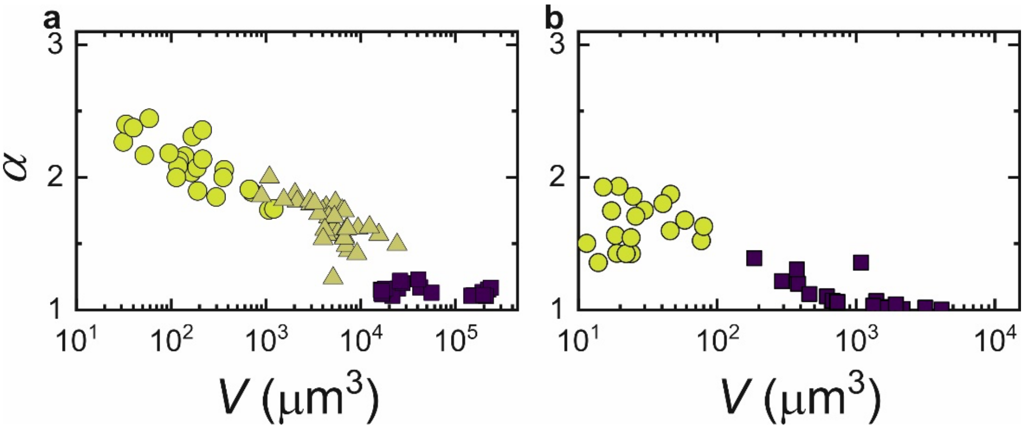

Supplementary Fig. 7 | Tactoidal phase diagram of BLG II and SCNC at equilibrium. The plots show the aspect ratio (α=R/r) versus volume (V) of the tactoids that are formed in the suspension with a concentration that is set within the isotropic–nematic coexistence region (Table S2). The circle, triangle, and square symbols show homogenous, bipolar, and cholesteric tactoids, respectively. The data at equilibrium are collected from the samples of BLG II (**a**) and SCNC (**b**) that are placed in a cuvette. For the equilibrium phase diagram of the BLG I, we refer to our recent study, where we see the transition from homogenous to bipolar at a volume 2,000 μm^3 and bipolar to cholesteric at a volume of 7,000 μm^3.

Supplementary Table 1

Supplementary Table 1 Material properties considered in the DNS method to simulate self-assembly.

Symbol	Material properties	SI units
$\boldsymbol{\xi}$	Coherence length	m
$\boldsymbol{p_\infty}$	Macroscopic pitch length	m
$\boldsymbol{\alpha_e}$	Elastic anisotropy	-
$\boldsymbol{L_\Phi}$	Coefficient of the concentration gradient	J/m
$\boldsymbol{L_{\Phi-Q}}$	Coupling coefficient of the concentration-orientation gradient	J/m
$\boldsymbol{M_\Phi}$	Mass mobility	m^5/sJ
$\boldsymbol{M_Q}$	Rotational mobility	m^3/sJ
$\boldsymbol{ck_BT}$	Thermal energy per unit volume of dispersion	J/m^3

Supplementary Table 2

Supplementary Table 2 Summary of proposed exponents, $R_{reg.}^2$ is computed based on regression analysis of the DNS results.

Phase	$\boldsymbol{n}$	$R_{reg.}^2$	$\boldsymbol{m}$	$R_{reg.}^2$	$\boldsymbol{p}$	$R_{reg.}^2$	$\boldsymbol{q}$	$R_{reg.}^2$	$\boldsymbol{-2p-q}$	$R_{reg.}^2$
HN	1	0.96	-0.5	0.96	-0.25	0.95	1	0.96	-0.5	0.91
BN	1	0.97	-0.5	0.92	-0.25	0.93	1	0.95	-0.5	0.45
N*	1	0.97	-0.5	0.95	-0.25	0.96	1	0.96	-0.5	0.40

Supplementary Table 3

Supplementary Table 3 Amyloid fibrils and cellulous nanocrystals suspensions properties obtained with experiments or calculated.

Sample	ϕ_I (wt%)	ϕ_N (wt%)	ϕ_I (vol%)	ϕ_N (vol%)	ξ (μm)
BLG I	2.0	2.5	1.5	1.9	1.3
BLG II	2.0	2.5	1.5	1.9	1.0
SCNC	2.4	3.5	1.6	2.3	0.9

Supplementary Note 1

Microfluidic system

To perform the relaxation experiments of the tactoids, we use a microfluidic system with contraction-abrupt expansions design (Supplementary Ref. 1-2), allowing to elongate the tactoids with different volumes and let them relax to the equilibrium (see Supplementary Figure 1). Tactoids with various volumes are formed inside the channel at upstream region. Tactoids travel in flow direction and are elongated as shown schematically in Supplementary Figure 1 in the elongation region (or contraction zone). Right after the elongation region, the tactoids get elongated again but in the perpendicular direction with respect to the flow direction and undergo relaxation to the equilibrium state in the relaxation region in Supplementary Figure 1. The main reason for the elongation of the tactoids at the beginning of the relaxation region is the high extension rate of the flow in y-direction in the expansion zone, i.e. $\dot{\varepsilon}_{yy} = \partial u_y / \partial y$, compared to the shear rate in the flow direction $\dot{\Gamma}_{xy} = \partial u_x / \partial y$. For U = 1.5 μm s^{-1} (where U is the flow speed in the straight channel before the extension zone) used in this study to understand the relaxation dynamics of the tactoids, we found the ratio of the $\dot{\varepsilon}_{yy}$ / $|\dot{\Gamma}_{xy}|$ to be always over 40 along the centerline of the channel (for details we refer the interested readers to our recent study in Supplementary Ref. 3).

Supplementary Note 2

Development of the analytical formula estimating the characteristic shape relaxation time, τ_s, in terms of material properties and tactoid's size

Based on an overdamped system with several frictional processes, we consider that the characteristic shape relaxation time, τ_{s}, is the sum of two contributions anisotropic and isotropic as

$$\tau_{\mathrm{s}} = \tau_{\mathrm{a}} + \tau_{\mathrm{i}}, \tag{1}$$

where τ_{i} is characteristic shape relaxation time of elongated isotropic tactoids. In the literature of simple droplets relaxation, τ_{i} has been well established as (Supplementary Ref. 4-5)

$$\tau_{\mathrm{i}} = \frac{\beta \mu_{\mathrm{N}} R_{\mathrm{equiv.}}}{\gamma}, \tag{2}$$

where γ is the interfacial tension, $R_{\mathrm{equiv.}} = ((r^2 R)^{1/3})$ is the equivalent radius of tactoids, μ_{N} the viscosity of the tactoids -taken to be equal to viscosity of the nematic phase- and $\beta =$

$\frac{(2\hat{\eta}+3)(19\hat{\eta}+16)}{40(\hat{\eta}+1)}$, where $\hat{\eta} = \frac{\mu_{\mathrm{N}}}{\mu_{\mathrm{I}}}$ is the ratio of viscosities of the nematic phase μ_{N} and that of the isotropic medium, μ_{I}. In Supplementary Eq. 1, τ_{a} describes the liquid crystalline anisotropic contribution due to the presence of orientational order, gradient elasticity, anisotropic viscoelasticity, rotational dissipation, and concentration gradients. In general, τ_{a} depends on two factors: material properties and tactoid's size meaning $\tau_{\mathrm{a}} \propto \Psi$ where Ψ encompasses the power laws describing the impact of material and size dependencies. The generic form of Ψ can be obtained through dimensional analysis of the governing equations of self-assembly. As can be appreciated from the modeling presented in this work, see Supplementary Ref. 6, there are eight material properties involved in the self-assembly. These material properties along with their SI units are summarized in Supplementary Table 1.

In Supplementary Table 1, c, k_{B}, and T represent the number density ($1/\mathrm{m}^3$), Boltzmann constant (J/K), and temperature (K), respectively. Note that $\alpha_e = \frac{L_2}{L_1}$, with L_1 and L_2 the Landau constants, and in the terms of Frank elastic constant, it becomes:

$$\alpha_{\mathrm{e}} = 2\frac{K}{K_2} - 1, \tag{3}$$

with K the Frank elastic constant for splay and bending (assumed to be equal) and K_2 the Frank twist elastic constant. Furthermore, to reveal the impact of the tactoid's size, the characteristic tactoid size $R_{\mathrm{equiv.}}$ is considered in the formulation of Ψ.

Before proceeding to carry out the dimensional analysis, we refine the selection of material properties to avoid irrelevant or redundant quantities. For tactoids that relax to nematic phases either homogenous or bipolar, the macroscopic pitch length, p_∞, does not affect τ_{a} as for nematic phases and $p_\infty = \infty$. In such cases, the p_∞ is an irrelevant material property and should be excluded from the dimensional analysis. In addition, Supplementary Ref. 7 has shown that, for cholesteric tactoids, the pitch length depends on the tactoid's size; hence, the p_∞ should also be excluded from the dimension analysis for tactoids that relax to cholesteric phases because of the presence of the characteristic tactoid size in the dimension analysis. In other words, the macroscopic pitch length, p_∞, should be excluded from the entire dimension analysis.

Applying dimensional analysis on the above-mentioned contributing factors yields

$$\Psi = \Upsilon \frac{1}{ck_{\mathrm{B}}T}\left(\frac{L_{\Phi-\mathbf{Q}}}{L_{\Phi}}\right)^{n} \alpha^{m} M_{\Phi}^{p} M_{\mathbf{Q}}^{-p-1} \; R_{\mathrm{equiv.}}^{q} \xi^{-2p-q}, \tag{4}$$

where Υ is a positive dimensionless constant and all exponents, i.e. n, m, p, and q, can be any real dimensionless number. For simplicity and without loss of generality, we consider $\Upsilon = 1$ while defining pre-factor b as $\tau_{\mathrm{a}} = \mathrm{b}\,\Psi$.

To determine the exponents present in Supplementary Eq. 4, an extensive uncorrelated parametric study for each phase of homogenous nematic (HN), bipolar nematic (BN), and chiral nematic (N*) is performed. Thereafter, the anisotropic contribution, $\tau_{\mathrm{a}} = \tau_{\mathrm{s}} - \tau_{\mathrm{i}}$, is computed using equations S1-S2 and fitted with the power laws shown in Supplementary Eq. 4. Through regression analysis, the proposed exponents are tabulated in Supplementary Table 2.As can be seen in Supplementary Table 2, for each of the following terms $\left(\frac{L_{\Phi-\mathbf{Q}}}{L_{\Phi}}\right)^{n}$, α^{m}, $M_{\Phi}^{p} M_{\mathbf{Q}}^{-p-1}$, and $R_{\mathrm{equiv.}}^{q}$, a unique exponent is found for different phases which results in excellent regression, $R_{\mathrm{reg.}}^{2} > 0.9$. Regarding the exponent of coherence length, the regression analysis of the DNS results suggests -0.5 for homogenous nematic phase, and -2 for bipolar nematic and cholesteric phases. However, to reach dimensional unit consistency, the exponent of the coherence length must be -0.5 for all three phases. Therefore, the DNS results underestimate the exponent of coherence length for bipolar and cholesteric phases. It should also be further noted that phase transition between phases depends on the material properties and the characteristic length. Investigation on material property and size dependencies of the phase transition is outside the present work's scope.

Substituting the exponents shown in Supplementary Table 2 into Supplementary Eq. 4, yields

$$\Psi = \frac{1}{ck_{\mathrm{B}}T} \frac{L_{\Phi-\mathbf{Q}}}{L_{\Phi}} \frac{R_{\mathrm{equiv.}}}{\alpha^{1/2} M_{\Phi}^{1/4} M_{\mathbf{Q}}^{3/4} \xi^{1/2}}. \tag{5}$$

Due to the fact that experimental measurements of $L_{\Phi-\mathbf{Q}}$, L_{Φ}, M_{Φ}, and $M_{\mathbf{Q}}$ are not available and certainly difficult to determine experimentally, we proceed to estimate them using liquid crystal physics. In terms of fundamental properties, according to Supplementary Ref. 8, $W \approx \frac{S(\Delta\phi) L_{\Phi-\mathbf{Q}}}{\sqrt{L_{\Phi}/ck_{\mathrm{B}}T}}$, where W, S, and $\Delta\phi$ are anchoring strength (J/m^2), uniaxial order parameter, and the volume fraction jump across the

interface, respectively. In view of $\gamma \approx \sqrt{ck_{\mathrm{B}}TL_{\phi}}$ (Supplementary Ref. 8) and the fact that $\frac{W}{\gamma} = \omega$, $\frac{L_{\phi-\mathbf{Q}}}{L_{\phi}}$ can be reformulated as

$$\frac{L_{\phi-\mathbf{Q}}}{L_{\phi}} \propto \omega. \tag{6}$$

The next step is to estimate the mobilities M_{ϕ} and $M_{\mathbf{Q}}$ in terms of fundamental physical properties. According to Supplementary Ref. 9, the rotational diffusion coefficient and translational diffusion coefficient are defined as, respectively, $\overline{D_{\mathrm{r}}} \propto \frac{k_{\mathrm{B}}T(2\ln(2L/D)-1)}{\mu_{\mathrm{N}}L^3}$ and $D_{\mathrm{G}} \propto \frac{k_{\mathrm{B}}T\ \ln(L/D)}{\mu_{\mathrm{N}}L}$ with SI units as $\mathrm{s}^{-1}$and m^2/s, respectively; here L and D represent the average fiber length and diameter, respectively. Furthermore, the mobilities can be found by $M_{\phi} = D_{\mathrm{G}}/ck_{\mathrm{B}}T$ and $M_{\mathbf{Q}} = \overline{D_{\mathrm{r}}}/ck_{\mathrm{B}}T$; and in consequence, their SI units become $\mathrm{m}^5/\mathrm{J.s}$ and $\mathrm{m}^3/\mathrm{J.s}$, respectively. Therefore,

$$M_{\phi} \propto \frac{\ln(L/D)}{c\mu_{\mathrm{N}}\mathrm{L}} \tag{7}$$

$$M_{\mathbf{Q}} \propto \frac{2\ln(2L/D)-1}{c\mu_{\mathrm{N}}L^3}. \tag{8}$$

Substitute Supplementary Eq. 3 and Supplementary Eq. 6 into Supplementary Eq. 5, the equality of $\tau_{\mathrm{a}} = \mathrm{b}\ \Psi$ can be expanded as

$$\tau_{\mathrm{a}} = \mathrm{b}\left[\frac{\omega}{ck_BT(2K/K_2-1)^{1/2}M_{\phi}{}^{1/4}M_{\mathrm{Q}}{}^{3/4}\xi^{1/2}}\right]R_{\mathrm{equiv.}}. \tag{9}$$

Note that each of Supplementary Eq. 6-8 can turn to be equality equations by use of constant pre-factors, and we can consider all these constants embedded in the main pre-factor b in Supplementary Eq. 9. Putting all together, characteristic shape relaxation time becomes:

$$\tau_s = \mathrm{b}\left[\frac{\omega}{ck_BT(2K/K_2-1)^{1/2}M_{\phi}{}^{1/4}M_{\mathrm{Q}}{}^{3/4}\xi^{1/2}} + \frac{\beta\mu_{\mathrm{I}}}{b\gamma}\right]R_{\mathrm{equiv.}}, \tag{10}$$

that predicts the characteristic shape relaxation time τ_{s} with a single fitting parameter b.

Supplementary Note 3

Determination of length and height distributions of liquid crystalline suspensions

We analyzed AFM images using FiberApp software (Supplementary Ref. 10) and obtained the length and height (or fibrils diameter assuming a cylindrical shape for the fibrils) distributions of the BLG II and SCNC fibrils as shown in Supplementary Figure 3. For details on the length and height distributions

of BLG I fibrils we refer to our recent study (Supplementary Ref. 3). The arithmetic mean length $L_{f,m}$, weighted mean length $L_{f,w}$, and mean arithmetic diameter (height) $D_{f,m}$ of each liquid crystalline system are reported in Table 1 in the main text. The fitting parameters of lognormal distributions fitted to data are as follow: BLG I: $\mu_{fitting} = 5.5 \pm 0$ and $\sigma_{fitting} = 0.6 \pm 0$ for the length distribution, $\mu_{fitting} = 0.9 \pm 0$ and $\sigma_{fitting} = 0.3 \pm 0$ for the height distribution; BLG II: $\mu_{fitting} = 5.2 \pm 0$ and $\sigma_{fitting} = 0.8 \pm 0$ for the length distribution, $\mu_{fitting} = 1.4 \pm 0$ and $\sigma_{fitting} = 0.2 \pm 0$ for the height distribution; SCNC: $\mu_{fitting} = 5.0 \pm 0$ and $\sigma_{fitting} = 0.5 \pm 0$ for the length distribution, $\mu_{fitting} = 1.5 \pm 0$ and $\sigma_{fitting} = 0.3 \pm 0$ for the height distribution.

Supplementary Note 4

Determination of amyloid fibrils and cellulose nanocrystals suspensions properties

Here we provide details on the calculation of the properties of the liquid crystalline systems used in this study, reported in Table 1 in the main text.

Effective diameter. We calculate the effective diameter as proposed by Onsager (Supplementary Ref. 11) taking into account the electrostatic interactions on the rods diameter. The effective diameter is obtained with

$$D_{\text{eff.}} = D + k^{-1}(\ln A + C + \ln 2 - \tfrac{1}{2}), \tag{11}$$

where k^{-1} is the Debye length, C is the Euler's constant that is 0.577 and A is given by (Supplementary Ref. 12)

$$A = \frac{8\pi Q e^{-kD}}{{A_c}^2 k^3 D^2 k_1^2(\frac{1}{2}kD)}, \tag{12}$$

with Q the Bjerrum length which is 0.70 nm, k_1 the modified Bessel function of the second kind and ${A_c}^{-1}$ the linear charge density. For amyloid fibrils (Supplementary Ref. 13-15), the linear charge density is 0.43 e/nm, Debye length is 2.6 nm^2 at ionic strength of 13.4 mmol/L. For sulfated cellulose nanocrystals (Supplementary Ref. 16), the linear charge density is 0.66 e/nm and the Debye length is 4.7 nm^2 at ionic strength 4.2 mmol/L. Putting all of the parameters together, we get the effective diameter values reported in Table 1.

Critical concentration. The critical concentrations are measured from the completely phase-separated suspension of the liquid crystalline with a concentration that is set within the isotropic–nematic coexistence region. In Supplementary Table 3, ϕ_{I} and ϕ_{N} denote the concentration of the isotropic and nematic phases of the phase-separated systems, respectively, that were measured gravimetrically. The volumetric concentration is calculated as

$$\phi\ (\text{vol\%}) = \frac{\phi\ (\text{wt\%})}{\phi(\text{wt\%}) + [1-\phi\ (\text{wt\%})]\rho_{\text{fibrils}}/\rho_{\text{water}}}, \tag{13}$$

where ρ_{fibrils} is the density of the fibrils that is 1.3 g/cm^3 for amyloid fibrils (Supplementary Ref. 17) and 1.5 g/cm^3 for cellulose nanocrystals (Supplementary Ref. 18.) The term ρ_{water} is the density of the water as the host fluid in BLG and SCNC liquid crystalline systems.

Elastic constants. We used analytical expressions previously proposed to calculate the splay, bend and twist elastic constants (Supplementary Ref. 19-20). The twist constant K_2 is calculated according to $K_2 = \frac{k_B T}{D}\left(\frac{\phi_{\mathrm{N}} L_p}{D}\right)^{1/3}$, and the bend constant K_3 is obtained with $K_3 = \frac{4}{\pi}\frac{k_B T}{D}\phi_{\mathrm{N}}\frac{L_p}{D}$, where L_p is the persistence length that is estimated to be 1.98 and 3.3 µm for BLG (Supplementary Ref. 21) and SCNC (Supplementary Ref. 15), respectively. Note that D is taken to be equal to $D_{\text{eff.}}$ and we take K_1 to be equal to K_3.

Anchoring strength. To obtain anchoring strength ω, we followed Wulff construction estimating $\omega = (\alpha/2)^2$ when anchoring strength is higher than one and $\omega = \alpha - 1$ when anchoring strength is less than or equal to one. Note that here α denotes the aspect ratio (R/r) of the homogenous tactoids in the equilibrium state.

Interfacial tension. To estimate the interfacial tension of the tactoids we use the universal scaling law as (Supplementary Ref. 22-23)

$$\gamma = \mathrm{f}\frac{k_{\mathrm{B}}T}{LD}, \tag{14}$$

where f is a constant equal to 0.3 (Supplementary References 7 and 22). Note that D and L are taken to be equal to $D_{\text{f,m}}$, and $L_{\text{f,w}}$, respectively.

Viscosity. We measured the viscosities of the isotropic and nematic phases of the phase-separated liquid crystalline suspensions. We estimate the viscosity of the tactoids to be equal to the viscosity of the nematic phase and the viscosity of the medium phase to equal the viscosity of the isotropic phase. We measured that the viscosities of the liquid crystalline phases are different depending on the shear rate, showing shear thinning behavior. In our calculations in this study, we take the zero shear viscosity values as reported in Table 1 (Supplementary Ref. 24).

Coherence length. We estimate the coherence length taking into account that the coherence length scale with the length of the fibrils $\xi \propto L$ and our recent measurements (Supplementary Ref. 25) on the coherence length for BLG and SCNC where for BLG with average length 652 μm the coherence length is measured to be 2.7 μm and for SCNC with average length 325 μm the coherence length is measured to be 1.75 μm.

Supplementary Note 5

Modeling of the deformation of the tactoids

Here we present modeling of the deformation of the tactoids under external stresses. This has been well documented for simple fluids, but for tactoids, the physics becomes complex due to the energy terms associated with the internal structure of the tactoids and their anisotropic features. We look at the deformation of the droplet under uniaxial flow field with extension rate given $\dot{\varepsilon}_{xx} = \frac{\partial u_x}{\partial x}$, where the u_x is the flow speed and x is the direction of the motion of the flow (see Supplementary Figure 1). Our approach relies on capturing the energy gained by the tactoids under the external stresses imposed by extensional flow field and incorporating that energy to free-energy landscape of the tactoids that is well described by scaling form of Frank–Oseen elasticity theory.

To start we consider a tactoid under an extensional flow that is at is elongated under a flow field shown in Supplementary Figure 1. To find the normal stresses applied to the tactoid, we first note from $\dot{\varepsilon}_{xx} = \frac{\partial u_x}{\partial x}$ that the velocity in x-direction is

$$u = \dot{\varepsilon}x + U, \tag{15}$$

where U is constant for a given volumetric flow rate. Supplementary Eq. 15 in the r'-z' coordinate, in Supplementary Figure 1, becomes

$$u = \dot{\varepsilon}(z' + \Delta x) + U, \tag{16}$$

where Δx is the distance between two coordinate systems of x-y and r'-z' in x (or z') direction. Having the velocity in x direction, from continuity equation the following holds

$$\frac{\partial u}{\partial z'} + \frac{1}{r'}\frac{\partial (r'v)}{\partial r'} = 0. \tag{17}$$

Therefore, from Supplementary Eq. 16-17, one can see that

$$v = -\frac{\dot{\varepsilon}}{2}r' + \frac{c_0}{r'}, \tag{18}$$

where the value of c_0 can be found using kinematic condition stating that the velocity is parallel to the droplet interface at interface, meaning

$$\left(\frac{d(r(z'))}{dz'}\right)_{r'=r(z')} = \frac{v}{u}. \tag{19}$$

From Supplementary Eq. 18 and the boundary condition in Supplementary Eq. 19, we calculate the term v as

$$v = -\frac{\dot{\varepsilon}}{2}r' + \frac{r(z')}{r'}\left[\frac{\dot{\varepsilon}r(z')}{2} + u\frac{d(r(z'))}{dz'}\right]. \tag{20}$$

The normal stresses at the interface of the tactoid are obtained as following

$$(\sigma_{r'r'})_{r'=r(z')} = 2\mu\left(\frac{\partial v}{\partial r'}\right)_{r'=r(z)} = -2\mu\left[\dot{\varepsilon} + \frac{u}{r(z')}\frac{d(r(z'))}{dz'}\right], \tag{21}$$

$$(\sigma_{z'z'})_{r'=r(z')} = 2\mu\left(\frac{\partial u}{\partial z'}\right)_{r'=r(z')} = 2\mu\dot{\varepsilon}, \tag{22}$$

where $\sigma_{r'r'}$ is the normal stress in r' direction and $\sigma_{z'z'}$ is the normal stress in z' direction. As stated in the main text, our analysis is for the case when the tactoids are under extreme deformation, this allows us to approximate a cylindrical shape for the tactoid under extension meaning $r(z') = r$, thus we have $\frac{d(r(z'))}{dz'} = 0$. This leaves us with $(\sigma_{r'r'})_{r'=r(z')} = -2\mu\dot{\varepsilon}$ from Supplementary Eq. 21. Next, we calculate the rate of the energy gained by the tactoid as

$$\frac{dE}{dt} = \int \boldsymbol{\sigma}.\mathbf{u}_i \, dS = \int \sigma_{r'r'} v_s \, dS_{r'} + \int \sigma_{z'z'} u_s dS_{z'}, \tag{23}$$

where we set the displacement velocity of interface v_s and u_s as $v_s = dr/dt$ and $u_s = dR/dt$. The terms $dS_{r'}$ and $dS_{z'}$ in capture the area of the tactoids in r' and z' directions, respectively, as $dS_{r'} = Rdr'$ and $dS_{z'} = rdr'$. Note that here volume of the tactoids kept unchanged during deformation as previously we have shown[3], $r^2R =$ constant resulting in $dR/dt = -2\frac{R}{r}dr/dt$. Substituting all parameters in Supplementary Eq. 23, we conclude the energy gained by the tactoid under extensional flow as

$$\frac{dE}{dt} = -6\mu_{\mathrm{I}} V \dot{\varepsilon} \frac{1}{r}\frac{dr}{dt} \tag{24}$$

where the pre-factor 6 may somehow vary without any loss of generality depending on the scaling assumptions taken (it becomes 8 for a perfect cylinder of radius *r* and length 2*R*). We now return to the free energy landscape of the tactoids, where the total free energy of the tactoid F_{E} is described in scaling form such as (Supplementary Ref. 7):

$$F_{\mathrm{E}} \sim \gamma R r \left[1 + \omega \left(\frac{r}{R}\right)^2\right] + KV\left(\frac{1}{R}\right)^2 + \frac{1}{2}K_2(\theta + q_\infty)^2 V \tag{25}$$

We propose that the rate of the energy gained by the tactoids due to the normal stresses from the flow field should be equal to the rate of the energy changes in the free energy landscape of the tactoids meaning $\frac{dE}{dt} = \frac{dF_{\mathrm{E}}}{dt}$, where

$$\frac{dF_{\mathrm{E}}}{dt} \sim 4\frac{Kr^3}{V}dr/dt - \gamma\frac{V}{r^2}dr/dt + 5\gamma\omega\frac{r^4}{V}dr/dt. \tag{26}$$

Additionally, as all three classes of the homogenous, bipolar and cholesteric tactoids hold homogenous configuration under extreme deformation, as can be seen in Figure 1 and our recent study (Supplementary Ref. 3), we ignore first term in Supplementary Eq. 26 meaning that the bulk elastic energy due to the splay and bending is set at zero. Additionally, the third term in Supplementary Eq. 25 is eliminated as, in the homogenous configuration and at constant tactoid volume, it does not change under deformation, so the rate of the energy gained by this term becomes zero. Setting $\frac{dE}{dt} = \frac{dF_{\mathrm{E}}}{dt}$, we have

$$5\gamma\omega r^6 + 6\mu\dot{\varepsilon}V^2 r - \gamma V^2 = 0, \tag{27}$$

giving us the steady-state elongated shape of the tactoids under a given extensional flow field.

Supplementary Note 6

Determination of the order parameter

The order parameter *S* is obtained using $S = \mathcal{r}/d\Delta n_0$ where $\mathcal{r}$ is the optical retardance value, d is the thickness of the sample and Δn_0 is the birefringence corresponding to a perfectly aligned nematic phase or when the order parameter is 1 (Supplementary Ref. 26-27). The retardance value $\mathcal{r}$ of every pixel within the tactoids is calculated using retardance images taken with LC-PolScope as $\mathcal{r}$ = (pixel gray value/max range of pixel gray value) × retardance ceiling. The value of *d* is calculated for every pixel assuming a spinodal shape for the tactoids (see the schematic provided in Supplementary Figure 5 where *d* for every pixel within the tactoid is calculated following $d = 2[a^2 - (dr)^2]^{1/2}$). Once we have $\mathcal{r}$ and *d* for every pixel, we calculate the average value as

$$(\mathcal{r}/d)_{\text{ave.}} = \frac{\sum_{n=1}^{n=\text{number of the pixels within the tactoids}} \mathcal{r}_i/d_i}{n}, \tag{28}$$

within the tactoid and take it as value of $(\mathcal{r}/d)_{\text{ave.}} = \frac{\mathcal{r}}{d}(t)$ for the tactoid at the given time during relaxation. We define

$$\mathcal{S} = \frac{S(t) - S_{\text{equil.}}}{S_{\text{init.}} - S_{\text{equil.}}} = \frac{\frac{\mathcal{r}}{d\Delta n_0}(t) - \frac{\mathcal{r}}{d\Delta n_0}_{\text{equil.}}}{\frac{\mathcal{r}}{d\Delta n_0}_{\text{init.}} - \frac{\mathcal{r}}{d\Delta n_0}_{\text{equil.}}} = \frac{\frac{\mathcal{r}}{d}(t) - \frac{\mathcal{r}}{d}_{\text{equil.}}}{\frac{\mathcal{r}}{d}_{\text{init.}} - \frac{\mathcal{r}}{d}_{\text{equil.}}}, \tag{29}$$

to capture the structural relaxation of the tactoids. As explained in the main text, we present our calculation independent from Δn_0 as an exact value of Δn_0 is often challenging to obtain experimentally, however is the same for a given liquid crystalline system. MATLAB programing is used to perform the measurements.

Supplementary Note 7

Nematic-cholesteric phase diagram of the tactoids at equilibrium

The nematic-cholesteric phase diagram of BLG II and SCNC at equilibrium are presented in Supplementary Figure 7. The data at equilibrium are collected from the samples that are placed in a cuvette. For the equilibrium phase diagram of the BLG I, we refer to our recent study (Supplementary Ref. 3).

Supplementary References

1. Cabral, J. T. & Hudson, S. D. Microfluidic approach for rapid multicomponent interfacial tensiometry. *Lab. Chip* **6**, 427–436 (2006).
2. Brosseau, Q., Vrignon, J. & Baret, J.-C. Microfluidic dynamic interfacial tensiometry (μDIT). *Soft Matter* **10**, 3066–3076 (2014) .
3. Almohammadi, H., Bagnani, M. & Mezzenga, R. Flow-induced order–order transitions in amyloid fibril liquid crystalline tactoids. *Nat. Commun.* **11**, 1-9 (2020).
4. Rallison, J. M. The deformation of small viscous drops and bubbles in shear flows. Annu. Rev. Fluid Mech. 16, 45–66 (1984).
5. Hudson, S. D., Cabral, J. T., Goodrum, W. J., Beers, K. & Amis, E. Microfluidic interfacial tensiometry. *Appl. Phys. Lett.* **87**, 081905 (2005).
6. Khadem, S.A. & Rey, A.D. Nucleation and growth of cholesteric collagen tactoids: A time-series statistical analysis based on integration of direct numerical simulation (DNS) and long short-term memory recurrent neural network (LSTM-RNN). *J Colloid Interface Sci.* **582**, 859-873 (2021).
7. Nyström, G., Arcari, M. & Mezzenga, R. Confinement-induced liquid crystalline transitions in amyloid fibril cholesteric tactoids. *Nat. Nanotech.* **13**, 330 (2018).
8. Das, S.K. & Rey, A.D. Magnetic Field-Induced Shape Transitions in Multiphase Polymer-Liquid Crystal Blends. Macromolecular Theory and Simulations. *Macromol. Theory Simul.* **15**, 469-489 (2006).
9. Doi, M. & Edwards, S. F. *The Theory of Polymer Dynamics*. Vol. 73 (Clarendon Press, 1988).
10. Usov, I. & Mezzenga, R. FiberApp: an open-source software for tracking and analyzing polymers, filaments, biomacromolecules, and fibrous objects. *Macromolecules* **48**, 1269-1280 (2015).
11. Onsager, L. The effect of shape on the interaction of colloidal particles. *Ann. NY Acad. Sci.* **51**, 627–659 (1949).
12. Vroege, G. J. & Odijk, T. Elastic moduli of a nematic liquid-crystalline solution of polyelectrolytes. *J. Chem. Phys.* **87**, 4223–4232 (1987).
13. Bagnani, M., Nyström, G., De Michele, C. & Mezzenga, R. Amyloid fibrils length controls shape and structure of nematic and cholesteric tactoids. *ACS Nano* **13**, 591–600 (2019).

14. Mezzenga, R., Jung, J. M. & Adamcik, J. Effects of Charge Double Layer and Colloidal Aggregation on the Isotropic-Nematic Transition of Protein Fibers in Water. *Langmuir* **26**, 10401–10405 (2010).

15. Bagnani, M., Azzari, P., De Michele, C., Arcari, M. & Mezzenga, R. Elastic constants of biological filamentous colloids: estimation and implications on nematic and cholesteric tactoid morphologies. *Soft Matter* **17**, 2158-2169 (2021).

16. Usov, I. *et al.* Understanding nanocellulose chirality and structure-properties relationship at the single fibril level. *Nat. Commun.* **6**, 7564 (2015).

17. Nyström, G., Fong, W. K., & Mezzenga, R. Ice-templated and cross-linked amyloid fibril aerogel scaffolds for cell growth. *Biomacromolecules* **18**, 2858-2865 (2017).

18. Nyström, G., Arcari, M., Adamcik, J., Usov, I. & Mezzenga, R. Nanocellulose fragmentation mechanisms and inversion of chirality from the single particle to the cholesteric phase. *ACS Nano* **12**, 5141-5148 (2018).

19. Odijk, T. Elastic constants of nematic solutions of rod-like and semi-flexible polymers. *Liq. Cryst.* **1**, 553–559 (1986).

20. Zhou, S., Cervenka, A. J. & Lavrentovich, O. D. Ionic-content dependence of viscoelasticity of the lyotropic chromonic liquid crystal sunset yellow. *Phys. Rev. E* **90**, 042505 (2014).

21. Adamcik, J. et al. Understanding amyloid aggregation by statistical analysis of atomic force microscopy images. *Nat. Nanotechnol.* **5**, 423–428 (2010).

22. van der Schoot, P. Remarks on the interfacial tension in colloidal systems. *J. Phys. Chem. B* **103,** 8804–8808 (1999).

23. Koch, D. L. & Harlen, O. G. Interfacial tension at the boundary between nematic and isotropic phases of a hard rod solution. *Macromolecules* **32**, 219–226 (1999).

24. Milliken, W. J., & Leal, L. G. Deformation and breakup of viscoelastic drops in planar extensional flows. *J. Non-Newton. Fluid Mech.* **40**, 355-379 (1991).

25. Khadem, S. A., Bagnani, M., Mezzenga, R. & Rey, A. Relaxation dynamics in bio-colloidal cholesteric liquid crystals confined to cylindrical geometry. *Nat. Commun.* **11**, 1-10 (2020).

26. Oldenbourg, R., Salmon, E. &Tran, P. Birefringence of single and bundled microtubules. *Biophys. J.* **74**, 645–654 (1998).

27. Gentry, B., Smith, D., & Käs, J.Buckling-induced zebra stripe patterns in nematic F-actin. *Phys. Rev. E* **79**, 031916 (2009).
