# Supplementary material for: Shape and structural relaxation of colloidal tactoids
Source: Nat Commun. 2022 May 19;13:2778. doi: 10.1038/s41467-022-30123-y (PMC9120485; doi:10.1038/s41467-022-30123-y)
Supplement: Supplementary file 3 — Description to Supplementary Information [file 41467_2022_30123_MOESM3_ESM.pdf]

Supplementary Movie 1 | An initially extended tactoid with volume 644 μm^3 undergoes shape relaxation while its configuration remains unchanged at homogenous configuration, corresponding to Fig. 1a.

Supplementary Movie 2 | An initially extended tactoid with volume 2,751 μm^3 relaxes both its shape and structure recovering a bipolar configuration upon relaxation, corresponding to Fig. 1b.

Supplementary Movie 3 | An Initially extended tactoid with volume 16,414 μm^3 relaxes to a cholesteric structure with three bands, corresponding to Fig. 1c.

Supplementary Movie 4 | Numerical simulation results capturing the relaxation of the initially extended tactoid to homogenous configuration, corresponding to Fig. 1a.

Supplementary Movie 5 | Numerical simulation results capturing the relaxation of the initially extended tactoid to bipolar configuration, corresponding to Fig. 1b.

Supplementary Movie 6 | Numerical simulation results capturing the relaxation of the initially extended tactoid to cholesteric configuration, corresponding to Fig. 1c
